# Pattern of gastrointestinal malignancies in a suburban centre in Southern Nigeria

**DOI:** 10.4314/ahs.v25i1.27

**Published:** 2025-03

**Authors:** Esteem Tagar, David Irabor, James Kpolugbo, Clifford Owobu, Ifeanyichukwu Chukwu

**Affiliations:** 1 Irrua Specialist Teaching Hospital, Surgery; 2 Ambrose Alli University College of Medicine, Surgery; 3 University College Hospital Ibadan, Surgery; 4 University of Ibadan College of Medicine, Surgery; 5 Irrua Specialist Teaching Hospital, Anatomic pathology; 6 Ambrose Alli University College of Medicine, Histopathology; 7 Irrua Specialist Teaching Hospital, Anaesthesia

**Keywords:** Gastrointestinal malignancies, colon, rectum, cancers, adenocarcinoma

## Abstract

**Background:**

Recent studies in sub–Saharan Africa have suggested an increasing incidence of gastrointestinal malignancies which consequently poses significant public health burden in terms of morbidity and mortality.

**Objective:**

This study was carried out to assess the distribution, clinical presentation, and histopathological characteristics of gastrointestinal malignancies in a tertiary health centre in Southern Nigeria.

**Methods:**

A retrospective review of all patients with histologic diagnosis of gastrointestinal malignancy in a tertiary health institution in Southern Nigeria between January 2013 and December 2022.

**Results:**

A total of 104 patients were included in the study. There were 64 males and 40 females with a male to female ratio of 1.6:1 and the peak age group was 41-50 years. The commonest sites affected were the colon and rectum (63.5%), followed by the stomach (22.1%). Adenocarcinoma was the predominant type of gastrointestinal malignancy, comprising 87.5% of the cases with most of them well differentiated. Others included sarcoma (6.7%), squamous cell carcinoma (1.9%), neuroendocrine tumour (1.9%), lymphoma (1%), and plasmacytoma (1%).

**Conclusion:**

Colon and rectal cancers were the predominant gastrointestinal malignancies with a male preponderance, and individuals between 41-50 years, who constitute the bulk of the country's workforce, were more affected. It is imperative to develop strategies aimed at reducing the incidence and fatality.

## Introduction

The gastrointestinal tract (GIT) consists of anatomically distinct segments, including the oesophagus, stomach, small intestine, colon, rectum, and anus^1^. In the GIT, just as there are regional variations in structure and function, so are the diseases^1^. The disorders could be congenital, inflammatory, or neoplastic (benign or malignant).

Globally, gastrointestinal tract malignancy accounts for 3.5 million (19.2%) of newly confirmed cancer cases and 2.2 million (22.7%) cancer related deaths.^2^ The more common primary GIT malignancies are oesophageal cancer, gastric cancer, and colorectal cancer accounting for 3.2%, 5.7%, and 10.0% of global cancers respectively, although with striking inter-regional variations^2^. Colorectal cancer ranks third and stomach cancer ranks fifth among all types of cancers worldwide, contributing 10.7% and 6.0% of the total number of new cases diagnosed in 2020^3^. Curiously, the small intestine is an uncommon site for tumour despite its great length and vast pool of dividing cells.

The pattern of primary gastrointestinal malignancy differs in different regions of the world. This is presumed to be determined by genetic, cultural practices, dietary, and socioeconomic factors^4^. Tumours arising from the mucosa of the stomach and intestines predominate over mesenchymal and stromal tumours. Adenocarcinoma constitutes 70% of all malignancies arising from the GIT^5^. The various histological types of tumours at different sites of the GIT also differ in their incidence and prognosis.

The incidence and prevalence of GIT malignancies are steadily increasing in Nigeria and the rest of Africa^6^. Colorectal carcinoma is the commonest malignancy of the GIT in Nigeria, with a relatively high incidence among the young and middle aged, adenocarcinoma being the predominant type^7^. The aim of this study was to assess the distribution, clinical presentation as well as histopathological characteristics of these malignancies at the Irrua Specialist Teaching Hospital (ISTH) within a period of ten years.

## Methods

### Study design and setting

This is a ten-year retrospective observational study carried out from January 2013 to December 2022 in the Department of Surgery at Irrua Specialist Teaching Hospital, (ISTH) Irrua in collaboration with the Department of Anatomic Pathology. The hospital is a tertiary care institution, located in Edo State, South-South Nigeria. It serves as a major referral centre from various parts of Edo and neighbouring states of Ondo, Kogi, and Delta.

### Inclusion and exclusion criteria

A total of 104 biopsies and resected specimens of the gastrointestinal tract with neoplastic lesions confirmed as malignant after histopathological examination were included in the study. Nineteen cases with incomplete information, inconclusive diagnosis, and cancers from the liver, gall bladder and pancreas were excluded.

### Data collection

A proforma was developed by the researcher and used to extract data from the departmental registers and the hospital records of patients who have been treated for malignant tumours of the gastrointestinal tract within the study period. Data collected include age, sex, clinical features, clinical diagnosis made by the unit consultant, location of tumour, type of specimen received, and histologic diagnosis. The specimens were from resected parts of the GIT and endoscopic biopsies. The histologic diagnosis for each patient was extracted from histology reports which had been written and verified by a consultant histopathologist after processing tissue biopsies at the time each patient was managed. All of the patients with gastrointestinal stromal tumours had their diagnosis confirmed through immunohistochemistry. The tumours were classified using the standard histological characteristics (according to the WHO classification system).

### Statistical analysis

The collected data were analysed using the Statistical Product and Service Solutions (SPSS) version 25 and presented in tables and figures.

## Ethical considerations

Institutional ethical approval was obtained from the Health Research Ethics Committee of ISTH prior to the commencement of this work. Its reference number is ISTH/HREC/20232310/502. All procedures were carried out in compliance with the 1964 Helsinki declaration and its later amendments. All data were fully anonymized.

## Results

A total of 104 cases of gastrointestinal malignancies seen between January 2013 and December 2022 with histological diagnosis and complete data were reviewed. There were 64 males (61.5%) and 40 females (38.5%) giving a male to female ratio of 1.6:1. (Table 1).

**Table 1:** Age and sex distribution of gastrointestinal malignancies

Age in Years	Number of Cases	Number of Cases
	Male (%)	Female (%)
≤ 20	1 (1.0)	0 (0.0)
21 – 30	3 (2.9)	4 (3.8)
31 – 40	9 (8.7)	3 (2.9)
41 – 50	17 (16.3)	8 (7.7)
51 – 60	12 (11.5)	11 (10.6)
61 – 70	14 (13.5)	7 (6.7)
71 – 80	7 (6.7)	5 (4.8)
≥ 81	1 (1.0)	2 (1.9)
Total (%)	64 (61.5)	40 (38.5)

The age group with the most GI malignancies was 41-50 years (25 cases, 24%). This was closely followed by 51-60 years (23 cases, 22.1%). Eighty-four (80.8%) of the patients were aged above 40 years while 20 (19.2%) were aged 40 years and below (Table 2).

**Table 2:** Age and site distribution of gastrointestinal malignancies

Age in Years	Stomach	Small Intestine	Colon	Rectum	Anus	Total
≤ 20	0	0	1	0	0	1
21 – 30	0	0	2	3	2	7
31 – 40	3	4	3	2	0	12
41 – 50	7	0	7	9	2	25
51 – 60	4	1	12	6	0	23
61 – 70	7	2	6	5	1	21
71 – 80	1	0	3	6	2	12
≥ 81	1	0	1	0	1	3
Total (%)	23 (22.1)	7 (6.7)	35 (33.7)	31 (29.8)	8 (7.7)	104 (100)

**Figure 1:**
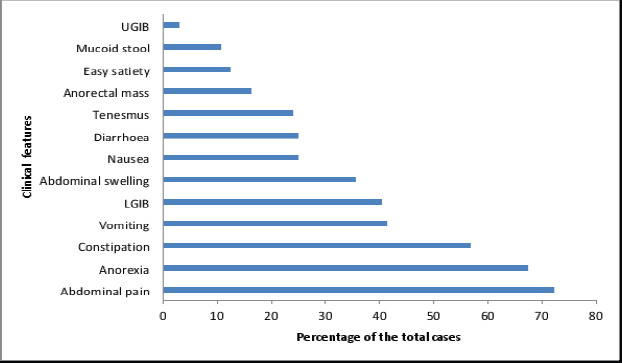
Clinical features on presentation

**Figure 2:**
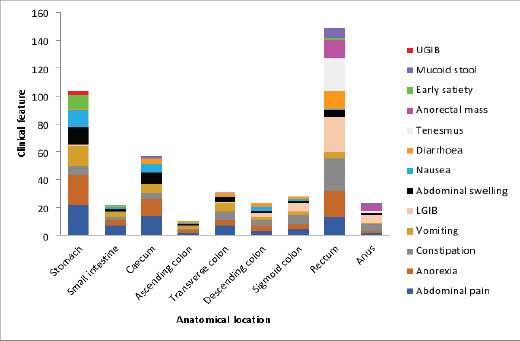
Anatomical distribution of clinical features of gastrointestinal malignancies

Most of the patients presented with abdominal pain (72.1%) and anorexia (67.3%). The other clinical features and their anatomical distribution are represented in figures 1 and 2 respectively.

**Figure 3:**
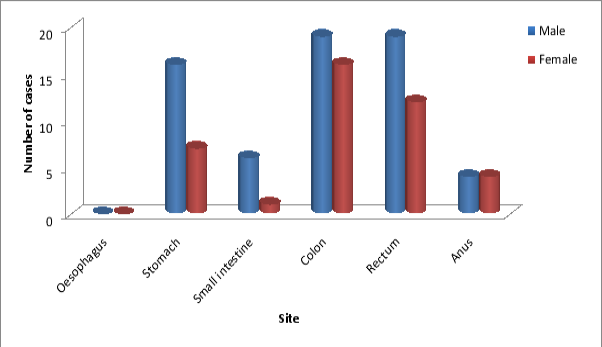
Site specific and sex distribution of gastrointestinal malignancies

Gastrointestinal malignancies were most common in the colon (33%), which was followed by rectum (29.8%), and stomach (22.1%). Anus (7.7%) and small intestine (6.7%) were the least commonly involved locations (Figure 3).

We did not record any case of oesophageal malignancy during the study period. The anatomical distribution of the malignancies in the various sites of the GIT is shown in table 3. Majority of the gastric cancers (82.6%) were in the antrum.

**Table 3:** Anatomical distribution of gastrointestinal malignancies in the GIT

Site		Frequency	Total (%)
Stomach	Cardia	2	23 (22.1)
	Body	2	
	Antrum	19	
Small Intestine	Duodenum	2	7 (6.7)
	Duodenojejunal area	1	
	Jejunum	2	
	Ileum	2	
Large Intestine	Colon	35	74 (71.2)
	Rectum	31	
	Anus	8	

**Figure 4:**
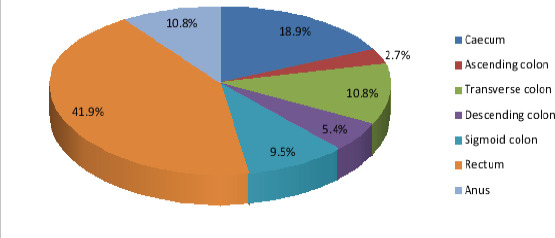
Anatomical distribution of large bowel cancers

Rectum (36%) was the commonest site of large bowel malignancy in this study, followed by the caecum (19%). The others are highlighted in figure 4.

Majority (89.4%) of cases were of epithelial origin while the mesenchymal tumours, lymphomas, neuroendocrine tumours and plasmacytoma constituted 10.6% (Table 4).

**Table 4:** Histological types of GI malignancies

Histological Type	Stomach	Small Intestine	Colon	Rectum	Anus	Total
Carcinoma	20	3	33	29	8	93
Sarcoma	2	1	2	2	0	7
Lymphoma	1	0	0	0	0	1
Neuroendocrine tumour	0	2	0	0	0	2
Plasmacytoma	0	1	0	0	0	1
Total (%)	23 (22.1)	7 (6.7)	35 (33.7)	31 (29.8)	8 (7.7)	104 (100)

Of the epithelial tumours (carcinomas), 91 (97.8%) was adenocarcinoma and 2 (2.2%) squamous cell carcinoma (SCC). The carcinomas were distributed in the colon, 33 (35.5%); rectum, 29 (31.2%); stomach, 20 (21.5%); anus, 8 (8.6%) and small intestine, 3 (3.2%).

The mesenchymal tumours (sarcomas) comprised gastric gastrointestinal stroma tumour (GIST) 2 (28.6%), small intestinal GIST 1 (14.3%), rectal GIST 1 (14.3%). Others were colonic leiomyosarcoma,1 (14.3%); rectal leiomyosarcoma,1 (14.3%) and colonic rhabdomyosarcoma,1 (14.3%), as shown in table 5.

**Table 5:** Distribution of histomorphology of various GI malignancies

Malignancy		Stomach	Small Intestine	Colon	Rectum	Anus	Total
Adeno Carcinoma	Well differentiated	8	0	21	10	1	40
	Moderately differentiated	1	1	10	9	1	22
	Poorly differentiated	9	2	2	9	3	25
	Signet ring type	2	0	0	0	0	2
	Mucinous type	0	0	0	1	1	2
Squamous Cell Carcinoma	Well differentiated	0	0	0	0	1	1
	Moderately differentiated	0	0	0	0		0
	Poorly differentiated	0	0	0	0	1	1
Plasmacytoma		0	1	0	0	0	1
MALToma		1	0	0	0	0	1
Alveolar Rhabdomyosarcoma		0	0	1	0	0	1
Leiomyosarcoma		0	0	1	1	0	2
GIST		2	1	0	1	0	4
Carcinoid		0	2	0	0	0	2
Total (%)		23 (22.1)	7 (6.7)	35 (33.7)	31 (29.8)	8 (7.7)	104 (100)

*GIST: Gastrointestinal Stromal Tumour

There were two cases of carcinoid tumour in the small intestine and a case each of gastric lymphoma and small intestinal plasmacytoma. Adenocarcinoma was the main type of GI malignancy in this study, comprising 87.5% of the cases, followed by GIST which constituted 3.8%. Of the 91 cases of adenocarcinoma, 40 (44.0%) were well differentiated, 22 (24.2%) were moderately differentiated while 25 (27.5%) were poorly differentiated. There were two (2.2%) cases of signet ring type in the antrum of the stomach and a case each of the mucinous type was recorded in the rectum and anus. Squamous cell carcinoma was found exclusively in the anus with a case each of well and poor differentiation.

## Discussion

The incidence and pattern of occurrence of gastrointestinal malignancies vary from region to region^4^,^6^. They demonstrate an array of histological patterns, an assortment of gross patterns, varied clinical presentations and an immense variability in their prognosis^8^. Gastrointestinal malignancies affected more males than females by a ratio of 1.6:1 in this study. This finding conforms to a male preponderance reported in most studies^4^,^8^,^9^. Gastrointestinal malignancies were seen over a wide range of age (18 years to 90 years) in this study. The highest distribution was found in the 5th decade, a relatively younger population, which is comparable to the age distribution reported by Parmar et al^8^.

However, the peak distribution in the studies by Dadhania et al. in India^9^ and Mahmoud et al. in Sudan^4^ was 6th and 7th decade respectively. Majority of our patients (80.8%) were aged above 40 years while 19.2% were aged 40 years and below. This compares favourably well with studies from Southern Nigeria^10^, Western Nigeria^6^, Sudan^4^, and India^8^. From the foregoing, it is apparent that the male patients, aged above 40 years are more affected in gastrointestinal malignancies. This interesting pattern may be a coincidental finding, or probably related to the cultural and social behaviour of some adult males with regards to engagement in certain risk factors associated with GI malignancies such as cigarette smoking and alcohol consumption.

Considering the clinical presentation of these patients, abdominal pain was the most common complaint (72.1%), followed by anorexia (67.3%), constipation (40.4%), vomiting (41.3%), haematochezia (40.4%), abdominal swelling (35.6%) as shown in figure 1. This finding is similar to that from another study done in Lagos, Nigeria^6^ which suggests that the clinical presentation is similar in gastrointestinal malignancies. Taking the anatomic location into account, GI malignancies have specific features which should trigger clinical suspicion.

For instance, gastric malignancy tends to present with abdominal fullness, recurrent vomiting, anorexia, and weight loss^6^. The commonest symptom of gastric cancer in the patients studied was upper abdominal pain which was present in 95.7%. Similar finding has been reported by other studies in Benin (82.6%)^11^ and Ibadan (75%)^12^. Most cases of gastric malignancies initially present with upper abdominal pain as the chief complaint, and treatment for peptic ulcer disease tend to offer some relief, especially in the early stages. This initial relief of pain is one of the reasons why most patients with gastric malignancies present late to the hospital^11^. Other presenting symptoms in decreasing frequency were anorexia, vomiting, abdominal swelling, nausea, and early satiety. Other studies on gastric cancer in Nigeria have reported similar findings, though at varying frequencies^11^,^12^. These symptoms should raise the suspicion of gastric malignancy in any patient.

In small bowel cancers, the presentation is usually silent until a complication like intestinal obstruction or sometimes intestinal perforation sets in. The symptomatic ones often present with nonspecific symptoms like abdominal pain, weight loss, nausea, vomiting, gastrointestinal bleeding (melena and haematochezia), diarrhoea with mucoid stool and palpable abdominal masses^13^. All the patients in our study presented with abdominal pain while 57.1% each presented with anorexia and vomiting. Other symptoms include constipation, abdominal swelling, nausea, and diarrhoea. Three patients (42.9%) presented with intestinal obstruction. Dabaja et al. in a single centre study reported abdominal pain in 66% of their patients and intestinal obstruction in 40% of patients which compares well with our study. They equally observed that bowel obstruction was more common in distally located tumours (jejunum and ileum) compared to duodenal tumours (47% vs 34%). Two of our cases (28.6%) were ileal while one (14.3%) was duodenal. Also, 24% of their patients had bleeding at the time of diagnosis, this we did not observe.

Right colonic cancers characteristically are exophytic cauliflower like lesions associated with insidious and chronic blood loss, thereby presenting with features of anaemia like weakness/fatigue. There may not be noticeable obstructive symptoms in the initial stage of right colonic cancers which cancers arising from the descending colon and rectosigmoid area are known for^6^. The prominent symptoms we observed in the cases of right colonic cancers were abdominal pain, anorexia, vomiting and abdominal swelling while for the left colonic cancers, constipation was more prominent followed by haematochezia.

The dominant symptom in rectal cancers was bleeding (80.6%). This was followed by tenesmus (74.2%) and constipation (74.2%). In a study conducted in Ibadan, Nigeria, by Irabor et al, bleeding per rectum was equally the commonest symptom in rectal cancers^14^. About 41.9% of the patients in our study had a palpable rectal mass which is lower than the 70% reported by Irabor et al. Bleeding, constipation, and tenesmus were the most common presenting features of anal cancer in our study which is consistent with a previous study in Ibadan^15^.

The commonest sites of GI malignancies among the patients studied were the colon and rectum in 66 cases (63.5%), followed by the stomach (22.1%). This concurs with many local and international studies, including the GLOBOCAN report, which observed that colorectal cancer takes the largest proportion of all types of gastrointestinal malignancies^2^,^5^,^6^,^10^. This is at variance with reports from Sudan and Togo where gastric cancer was the leading GI malignancy^4^,^16^. These geographic variations may be related to differences in risk factors, such as the prevalence rate of infection with Helicobacter Pylori (a bacterium with carcinogenic properties) in Sudan and Togo^4^,^16^ and accessibility to diagnostic means. Across Europe and United States, colorectal cancer has consistently been among the top three cancers^2^,^6^,^10^. It has been observed that there is a gradual increase in the trend in Africa^10^. Our observation of high proportion of colonic and rectal cancers in this study also suggests a rising incidence of the condition among Nigerians. This increase could be attributed to the fact that some African citizens have adopted the Westernized lifestyle including the ingestion of low fibres and high fat diets, and cigarette smoking^10^. In our study, anal cancer occurred in eight cases (7.7%) which is similar to the findings of Habeebu et al. (6.6%)^6^ and Abudu et al. (7.5%)^10^. Cases of small bowel cancer were equally few (6.7%).

Similar reports in this region have also shown that small bowel cancers are generally uncommon^4^,^6^,^17^. Not a single case of oesophageal cancer was found in our study. This could possibly be explained by the variability of predisposing factors among different populations. It could also be that the patients were referred to a neighbouring tertiary health institution from our general outpatient department due to unavailability of thoracic surgery for most of the time. This may be particularly so because the University of Benin Teaching Hospital in Benin, Edo State, which is about 87 km from Irrua saw 76 patients with oesophageal cancer over 11 years spanning the period from January 2004 to December 2014^18^.

Adenocarcinoma was the predominant histologic subtype of GI malignancy (87.5%) in this series. This is in consonance with previous studies in Nigeria. About 85.1% was observed in Uyo^10^, 89.6% in Lagos^6^, and 94.1% in Delta state^17^. Equal or slightly higher findings have been reported from West Africa, Europe, and Asia^16^,^19^. These findings are consistent with the histogenesis of the GIT, most of which are glandular tissues. Sarcoma, squamous cell carcinoma, carcinoid tumour, lymphoma, plasmacytoma were other histologic subtypes seen in this study; these are comparable with other studies, though reported in varying proportions^6^,^10^,^16^,^19^.

There were two peak age groups for stomach malignancies in this study: 41-50 years, and 61-70 years, both were of similar histologic types. While the former compares well with the study of Parmar et al.^8^, the latter is comparable with the study of Abudu et al.^10^ In contrast, Mahmoud et al.^4^ and Balekuduru et al.^20^ observed a peak age group of 51-60 years. Among the gastric tumours, adenocarcinoma with 20 (87%) cases was the predominant histologic type observed followed by malignant GIST 2 (8.7%) and MALToma 1 (4.3%). This conforms with the study of Purohit et al.^21^ which among 11 cases of stomach cancer observed that 8 (72.72%) were adenocarcinoma, 2 (18.18%) were malignant GIST and 1(9.09%) was non-Hodgkin's lymphoma. In the work of Parmar et al. 66.67% cases were adenocarcinoma, 16.67% cases were GIST, and 16.67% cases were diffuse large B cell lymphoma. Out of 20 cases of adenocarcinoma in our study, two cases (10%) belonged to the signet ring variety. The gastric MALToma was the only case of lymphoma in our series, and similar reports in this region have equally shown the rarity of lymphoma^6^,^10^,^17^.

In our case, the diagnosis was first considered after an abdominal ultrasound scan report suggested abdominal lymphoma. The patient then had an axillary lymph node biopsy performed with the histology revealing sinus hyperplasia. The diagnosis of MALToma was eventually confirmed through the histology of her upper gastrointestinal endoscopic biopsy.

We found adenocarcinoma to be the predominant tumour in the small intestine (42.9%). Purohit et al.^21^ found 50% cases of adenocarcinoma. However, in the study by Uchendu et al.^17^, sarcomas (GIST) were the predominant small intestinal cancers (80%) while we observed just 14.3% cases of GIST in our study. Carcinoid tumour is commonly seen in the small intestine and appendix. We found two cases (1.9%) in the small intestine. Similarly, Purohit et al.^21^ recorded all their three cases (3.1%) in the small intestine. Balekuduru et al.^20^ on the other hand observed one case (1.1%) in the small intestine and two cases (2.2%) in the stomach. A relatively low rate of small intestine cancers has been a general observation despite being the largest part of the GIT^17^. This paradox may be attributed to the short transit time of food particles, low bacterial load, high IgA concentration and less exposure to carcinogens.

The colon was the commonest site of all GI malignancies in this study with 35 cases (33.7%). Our cases were predominantly adenocarcinoma (94.3%). The peak age group of colon cancer was 51-60 years with a male to female ratio of 1.2:1. This is consistent with previous studies in Nigeria^22^ and india^20^. The peak age incidence of rectal cancer of 41-50 years in our study is comparable to the observation in India but a decade lower than was observed in Ibadan, Nigeria^22^. This may further buttress the global concern of an increasing incidence of rectal cancers in patients under 50 years of age and highlights the need to investigate potential causes and external influences such as lack of screening and behavioural factors. Majority of the cases in the rectum were also adenocarcinoma (93.5%) with a single mucinous variant.

Typically, anal cancer may be adenocarcinoma or squamous cell carcinoma in line with the normal epithelium of the proximal and distal 3rd of the anal canal.^1^ We observed 8 (7.7%) cases of anal cancer, 6 (75%) were adenocarcinoma with one mucinous variant and 2 (25%) were squamous cell carcinoma. This finding of adenocarcinoma being the predominant histopathological subtype is consistent with that of Irabor et al^15^. Parmar et al.^8^ reported 4 cases of anal cancers, all being adenocarcinoma with one mucinous type. On the contrary, all two cases reported by Uchendu et al.^12^ were SSC. Squamous cell carcinoma was also more common (85%) in a study in South Africa, and this histological type has been shown to have poorer prognosis^23^. The departure from the hitherto SCC dominance in our setting implies that only a few patients will benefit from chemo-radiation, with the majority benefitting from abdominoperineal resection. Although, a quite significant proportion of our patients may still decline surgical treatment because of cultural aversion to permanent colostomy^15^.

The most common type of histological differentiation of carcinoma seen overall in our study was the well-differentiated variant (44.1%). We however observed 50% of the poorly differentiated type in the anus with one case of mucinous variant. Higher overall incidence of well differentiated carcinoma (42%) was also observed by Balekuduru et al.^20^ This fact presents better prognostic implications.

## Conclusion

Gastrointestinal malignancies show a wide variation in the distribution, clinical presentation, and histopathological characteristics. Understanding their patterns and having a higher index of suspicion would ensure early detection, effective management, and possibly improve the outcome. The peak age distribution was in 41-50 years, a slightly younger age group with a male preponderance.

Abdominal pain and anorexia were the most common complaints (72.1% and 67.3% respectively). These complaints should not be dismissed in a hurry especially in patients above 40 years of age. We found majority of our cases (63.5%) in the colorectal region with adenocarcinoma being the predominant histologic subtype in 87.5% of the cases. Most of the carcinoma were well-differentiated (44.1%) which represents a better prognostic implication. Although, half of the patients with anal carcinoma showed poor differentiation which portends a poorer outcome. This calls for a high index of suspicion and prompt interventions in patients with anal symptoms.

The significant involvement of individuals who constitute the bulk of the country's workforce points at the need to make endoscopic equipment and training available to all tertiary centres in Nigeria to facilitate screening programs, improve diagnosis and hopefully reduce the rising incidence of gastrointestinal malignancies.

## Recommendation

The results and observations from the study will provide a valuable baseline information regarding frequency and pattern of gastrointestinal malignancies in our region. Further research into the peculiarities of the risk factors faced by Nigerians which has resulted in increased frequency of GI malignancies need to be done to have a better understanding of the changing pattern.

## Figures and Tables

**Figure 5A:**
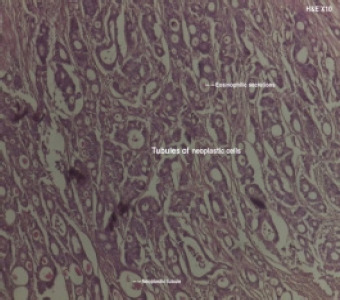
Colorectal adenocarcinoma

**Figure 5B:**
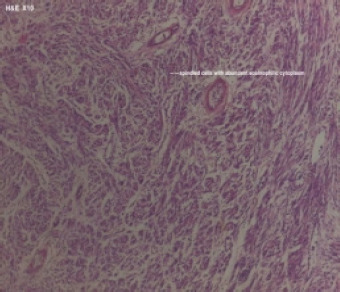
Gastrointestinal stromal tumour

**Figure 5C:**
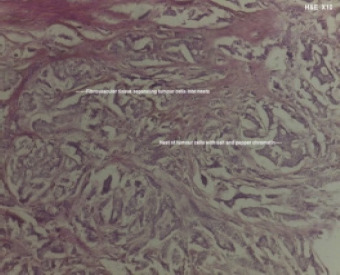
Carcinoid tumour

**Figure 5D:**
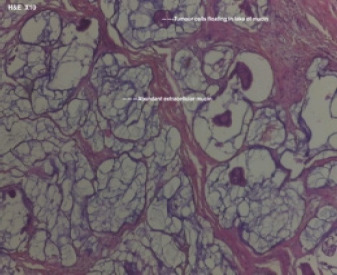
Mucinous carcinoma
